# Consumer Satisfaction and Efficacy of the Hangover Cure After-Effect^©^

**DOI:** 10.1155/2012/617942

**Published:** 2012-07-18

**Authors:** J. C. Verster, O. Berthélemy

**Affiliations:** ^1^Division of Pharmacology, Utrecht Institute for Pharmaceutical Sciences, Utrecht University, Universiteitsweg 99, 3584 CG Utrecht, The Netherlands; ^2^Deenox SAS, 86 rue de Paris, 91400 Orsay, France

## Abstract

A consumer satisfaction study was conducted to examine the effectiveness on hangover of After-Effect^©^, a new food supplement dedicated to improve well-being after an occasion of alcohol consumption. *N* = 113 persons were invited to participate in a home-based open label study to test the effectiveness of After-Effect^©^. On a night when they intended to consume alcohol, three pills were taken before alcohol consumption and two pills afterwards, before going to bed. The following day, participants completed a survey on the amount of alcohol consumed, hangover symptom severity, and satisfaction of the product. *N* = 103 participants completed the study. 88% of participants reported After-Effect^©^ to be effective in reducing alcohol hangover. After-Effect^©^ significantly improved overall hangover severity, and all individual hangover symptoms, except for palpitations. In addition, a significant reduction (*P* = 0.0001) in the severity score on concentration problems was reported when using After-Effect^©^. No gender differences were observed, and there was no relationship with the number of alcoholic drinks that were consumed. Consumers were satisfied with the product. In conclusion, consumer satisfaction and hangover severity scores suggest that After-Effect^©^ may be effective in reducing alcohol hangover. However, controlled, double-blind clinical trials should confirm these findings.

## 1. Introduction

Alcohol hangovers are the most commonly reported negative consequence of heavy drinking. About 80% of drinkers acknowledge having experienced a hangover at least once during the past year [1], a finding that is corroborated by clinical trials indicating that around 20% of drinkers are resistant to hangover [2]. Alcohol hangovers are characterized by a feeling of general misery, and several symptoms such as headache, thirst, sleepiness, and concentration problems are commonly reported [3].

The aftereffects of alcohol consumption experienced during hangover are often qualified as unpleasant and disabling. For example, subjects report missing classes, work, or other obligations due to hangovers, but also feelings of regret and mood changes may be the result of excessive alcohol consumption [3]. Hence, there is a clear need for a treatment or cure that prevents or reduces hangovers. On the Internet, many cures are marketed, but a systematic literature search revealed that the efficacy of the vast majority of them has not been scientifically investigated [4, 5]. Up to now, most potential hangover cures have shown no effectiveness, whereas other cures reduced only some of the core symptoms of alcohol hangover. For example, tolfenamic acid reduced severity scores of headache and nausea but had no effect on being tired [6]. Also, *Opuntia ficus indica* significantly reduced nausea, lack of appetite, and dry mouth but did not reduce complaints of headache, weakness, and dizziness [7].

The main reason for the absence of an effective hangover cure is that limited research has been devoted to elucidate the pathology of alcohol hangover [8]. The research that has been conducted shows that alcohol hangover is not simply the equivalent of dehydration, but that other mechanisms, such as activation of the immune system, may play a role in the genesis of alcohol hangover [8–10]. The partial improvement observed for tolfenamic acid (which inhibits prostaglandin synthesis) and *Opuntia ficus indica* (which is thought to reduce the inflammatory response to stressful stimuli) supports a potential role of the immune system in the development of alcohol hangover symptoms. However, much more research is needed to understand the pathology of alcohol hangover and develop an effective treatment [3].

Ethical concerns have been expressed concerning alcohol hangover research. For example, it has been argued that development of effective treatments for hangovers will result in increased alcohol consumption, due to the diminished negative consequences. There is, however, no scientific proof to support this assumption. Moreover, research showed that people generally do not adjust their drinking behavior after having experienced hangovers [11].

**Figure 1:**
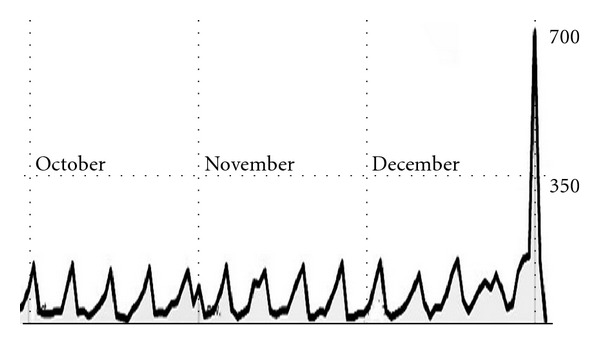
Number of visits on a French website for alcohol hangover (http://gueuledebois.info/). Data are shown from October 1st 2011 to January 2nd 2012. Each peak corresponds to a Sunday. Note the large peak at New Years day. The peak at November 1st corresponds to the day after Halloween (31 October). Data were obtained via Google Analytics.

For ages alcohol has been consumed by mankind, and the presence of hangovers was already reported more than 3000 years ago in ancient India. The Suśruta Samhitā, one of the oldest *Ā*yurvedic medicinal writings, refers to “*paramada*” when discussing alcohol hangover and reports on common hangover symptoms such as pain in the head and joints, loss of taste, and thirst [12]. Alcohol hangovers have been reported ever since throughout history, and as long as alcohol consumption is allowed, it is unrealistic to assume that any behavioral intervention will prevent hangovers from happening. Statistics from a French website on alcohol hangovers (http://gueuledebois.info/) confirm the need for information about hangovers and how to treat them.  Figure 1 gives an overview of the daily number of visits of the website during a 3 months period.

Each peak in the number of page views in Figure 1 corresponds to a Sunday. This is not surprising, given that the weekends, and especially Saturday evenings, are the most likely occasions of heavy drinking, which may result in a hangover the following day.

Although most people consume alcohol in moderation and do not regularly experience a hangover, the socioeconomic consequences of having a hangover are high [13]. That is, absenteeism and presenteeism are common consequences of having a hangover, and reduced productivity and increased risk of injury when operating dangerous machinery may be the result [14–16]. Also, while driving or flying when having a hangover, people put not only themselves at risk but also those who are surrounding them [17]. Hence, there are a number of arguments that plea for development of an effective cure that reduces or prevents alcohol hangover effects.

**Figure 2:**
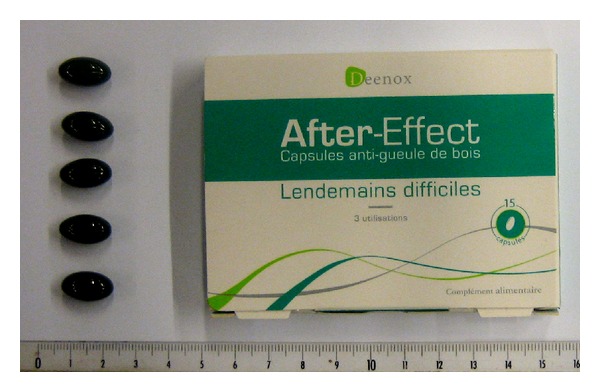
After-Effect^©^: package and capsules. Three capsules should be taken before alcohol consumption and two additional capsules before going to bed.

After-Effect^©^ is an example of such a newly developed hangover cure (see Figure 2). The product is currently sold in pharmacies by Deenox in France, and like many hangover cures it can also be ordered online. Instructions for using After-Effect^©^ are to take three capsules before alcohol consumption and 2 capsules after drinking, before going to bed. The ingredients of After-Effect^©^ comprise borage oil (gamma linolenic acid), fish oil (omega-3), vitamins B1, B6, and C, magnesium, *Silybum marianum* (silymarin), and *Opuntia ficus indica*. The rationale for the manufacturer to include these ingredients in After-Effect^©^ was based on the current available literature on hangover cures and their effectiveness in reducing hangover symptoms and on their potential mechanisms of action. Regarding *Opuntia ficus indica*, it should be noted that After-Effect^©^ contains a polar extract, which is different from the apolar extract used by Wiese et al. [7]. It is therefore unknown whether After-Effect^©^ will have similar beneficial effects on hangover such as described by Wiese et al. (i.e., reduced scores on nausea, dry mouth, and lack of appetite).  Table 1 summarizes the ingredients, suggested mechanism of action, and the corresponding hangover symptoms that showed to benefit from their use [18–29].

Table 1 reveals that there is scientific support showing that the individual ingredients of After-Effect^©^ can reduce several common hangover symptoms. However, their combined effect (i.e., the After-Effect^©^ formula) has not been scientifically investigated. Therefore, the objectives of the current study were to (1) examine the effectiveness of After-Effect^©^ and (2) to evaluate consumer satisfaction of this hangover aid. The design of the study followed a naturalistic approach [30, 31], which is quite common for consumer satisfaction studies [32]. Participants consumed alcohol at a place, quantity, and time of their own preference without interference of the researchers. On that occasion, they also used After-Effect^©^ and completed a questionnaire the following day.

## 2. Methods

A total of 113 persons were contacted by telephone to participate in the study. Participants were selected randomly among consumers that were registered in the panel of the consumer testing laboratory TechniSens. If they agreed to participate after the telephone contact, they received After-Effect^©^, instructions for use and the survey by regular mail. Informed consent was obtained from all subjects. The study took place on 1–15 September, 2010. Subjects were instructed to use After-Effect^©^ in an evening when they expected to consume a sufficient quantity of alcohol to leave them feeling unpleasant the following day. In that evening, participants were instructed to take 3 capsules of After-Effect^©^ before drinking and 2 capsules at bedtime. They were instructed to swallow the capsules with a glass of water and not to chew them. A survey was completed the day after the drinking session, as soon as they presumed their blood alcohol level had returned to zero. The completed surveys were returned by email to TechniSens.

In addition to demographics and information on the amount of alcohol consumed, the survey included questions about the specific hangover symptoms experienced. Hangover symptoms and their severity were scored using the acute hangover scale (AHS) [33]. Eight hangover symptoms (thirst, being tired, headache, dizziness, loss of appetite, stomachache, nausea, and heart racing) and total hangover severity were scored on a scale ranging from 0 (absent) to extreme (10). The mean of these nine scores is the overall hangover severity score. Similar to the items of the AHS, “concentration problems” was added as extra item since it is commonly experienced [34] and provides useful information on the potential impact of alcohol hangover on next-day performance. The items were scored after using After-Effect^©^. Participants acted as their own control. The scores obtained with After-Effect^©^ were thus compared to individual expected scores if After-Effect^©^ had not been taken with the same consumption of alcohol.

Finally, several consumer satisfaction questions were asked concerning the packaging of After-Effect^©^, the instructions for usage of the product, its perceived effectiveness and adverse events, and whether they would recommend the product to family and friends.

### 2.1. Statistical Analysis

Subjects that vomited in the evening when using After-Effect^©^ were excluded from the statistical analyses. Statistical analyses were performed using SPSS 19.0. Mean (SD) scores on the hangover items and the overall AHS score were computed. Symptom severity when using After-Effect^©^ and a regular hangover night was compared using paired sample *t*-tests. Scores of those who reported After-Effect^©^ to be effective or ineffective were compared using the same test. Percentages of endorsed items (% agreed versus % disagreed, or % effective versus % ineffective) were compared using a binominal test for proportions. Results were significant if *P* < 0.05.

## 3. Results

A total of 113 subjects participated in the study. Ten were excluded from the statistical analyses because they reported vomiting in the evening when they consumed alcohol and used After-Effect^©^. 103 subjects (21% men and 79% women) completed the study. Half of them were 25–30 years old, 25% were 31–35 years old, and 25% were 36–40 years old. In the evening out, 44% consumed 4–6 alcoholic consumptions, 46% consumed 7–9 alcoholic consumptions, and 10% more than 10 alcoholic drinks. Hangover symptom severity, with and without using After-Effect^©^, is summarized in Table 2.

**Figure 3:**
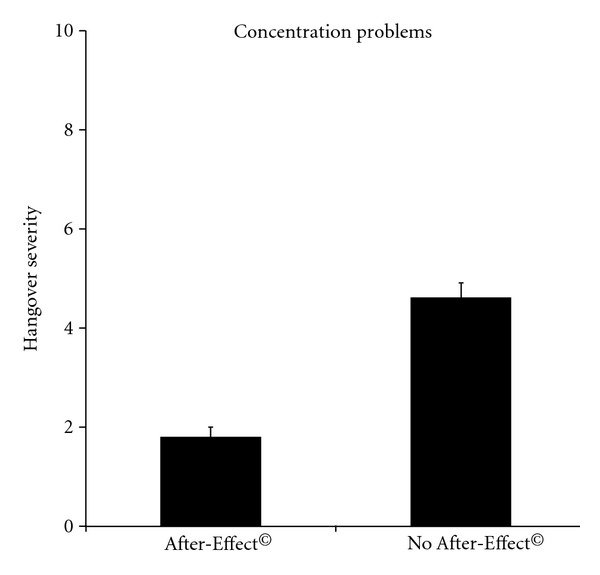
Severity of concentration problems with and without using After-Effect^©^.

**Figure 4:**
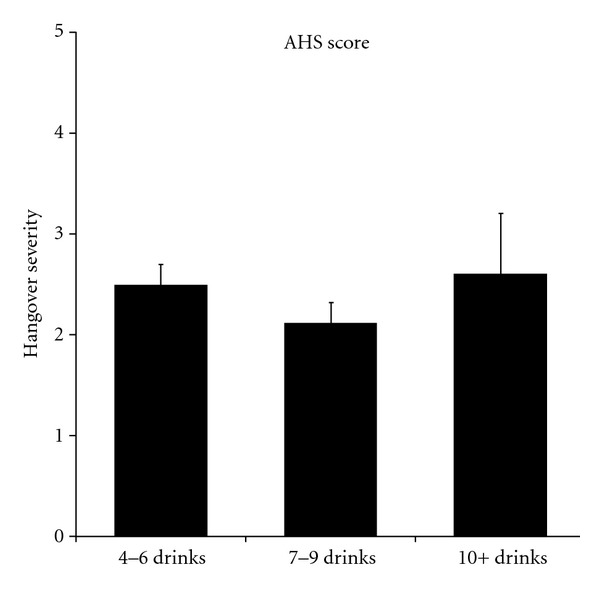
Overall hangover severity and total alcohol consumption.

It is evident from Table 2 that After-Effect^©^ significantly improved both overall hangover severity and individual hangover symptoms. In addition, a significant reduction (*P* = 0.0001) in the severity score on concentration problems was reported when using After-Effect^©^ (see Figure 3). No gender differences were observed, and there was no relationship with the number of alcoholic drinks that were consumed (see Figure 4).

The vast majority of consumers agreed (56%) or strongly agreed (32%) that After-Effect^©^ reduced the uncomfortable feeling usually experienced the day after consuming alcohol. A minority of 12% was not satisfied with the product's effectiveness. Of them, six persons (6%) reported that the product did not work in a free comment area of the questionnaire.  Table 3 summarizes the consumer satisfaction on how well After-Effect^©^ counteracts individual hangover symptoms.

Binominal tests (% agreed versus % disagreed) confirm that for each hangover symptom, except increased heart rate, After-Effect^©^ significantly more often produced a favorable effect than an unfavorable effect.

In those who reported that After-Effect^©^ was not effective, the overall hangover severity score and all scores of individual items except heart racing were significantly higher compared to scores of consumers who reported that After-Effect^©^ was effective (see Table 3). Although no effectiveness was reported by these subjects, After-Effect^©^ did significantly (*P* < 0.05) reduce their scores on being tired, headache, stomach ache, nausea, and the overall AHS hangover score (*P* = 0.002).

In 96% of subjects, the use of After-Effect^©^ caused no adverse effects. Nausea (2%) and bloating (1%) were reported as adverse effects of using After-Effect^©^, and one person (1%) stated the capsules to be too large to swallow.

On average, consumers were satisfied with the size of the package (mean score: 7.75 out of 10), the design of the package (mean score 7.69 out of 10), and the way it opens (mean score: 7.76 out of 10). About half of the subjects (52%) preferred the product to be taken as intended (3 capsules before drinking and 2 thereafter), whereas 48% preferred to take all 5 capsules before the evening out. The vast majority (84%) of participants acknowledged that they would recommend After-Effect^©^ to family or friends.

## 4. Discussion

The results from this open-label study suggest that After-Effect^©^ is likely to reduce the presence and severity of alcohol hangover symptoms. Consumer satisfaction scores confirm these findings. The significant reduction in concentration problems after using After-Effect^©^ is promising, because this may have a positive impact on cognitive and psychomotor impairment that is generally seen during alcohol hangover.

In contrast to other hangover cures that have been investigated, After-Effect^©^ shows to be effective in significantly reducing both overall hangover severity and scores on individual hangover symptoms. This underscores the rationale used in the development of After-Effect^©^ in combining those ingredients that have shown effectiveness in previous hangover studies. It can be speculated that the anti-inflammatory and antioxidative properties of the ingredients are responsible for the reduction in hangover symptom severity. However, from this consumer survey it cannot be established whether the immune system plays a vital role in the pathology of alcohol hangover symptoms and if the proposed mechanism of action of After-Effect^©^ is indeed responsible for the reported effectiveness of this hangover treatment.

There are a number of limitations of this study that should be addressed. A major limitation of the current study is that no placebo hangover treatment was included. With the current study design it therefore remains unsure if the reduction in hangover (symptom) severity can be ascribed to After-Effect^©^. Participants knew beforehand they were going to use After-Effect^©^. The hangover symptom scores obtained in that evening were then compared with retrospectively assessed scores for an evening with similar alcohol consumption, but without using any hangover treatment. This study design may have biased the outcome of the study because participants may have certain expectancies about the efficacy of After-Effect^©^ in reducing hangover symptoms. Therefore, it can equally be true that the reported improvements are in fact a placebo effect and not due to any efficacy of After-Effect^©^. The likelihood of this possibility is however small, given the large and consistent improvement that was reported on almost all hangover symptoms. Nevertheless, future research should be double-blind and include a drinking session with placebo After-Effect^©^. This will allow a more objective comparison with a drinking session on which no hangover cure is used than the comparison that was made in the current study, that is, a comparison with expected feelings if After-Effect^©^ had not been used. It would have also been interesting to test participants after a placebo alcohol session with and without administering After-Effect^©^ because hangover symptoms may in fact be “general” symptoms that are always experienced by participants, also without consuming alcohol. In addition, this would enable a more valid examination to determine if After-Effect^©^ itself causes any adverse effects than how this was assessed in the current study. Future research should address these issues.

The fact that this was a naturalistic study is sometimes also considered as a limitation. However, there are both advantages and disadvantages of using a naturalistic design instead of a controlled study [3]. Controlled clinical trials enable researchers to standardize various factors that may influence the presence and severity of alcohol hangover symptoms such as beverage type, drinking speed, sleep time, activities (e.g., dancing), smoking, and food consumption. Yet, if one aims to mimic a real-life drinking situation, the naturalistic approach seems best. Despite the fact that many issues are uncontrolled in naturalistic studies, consumer satisfaction ratings have shown to be more reliable when obtained in a real-life setting [32], that is, drinking alcohol in a bar and sleeping and having a hangover at home. In fact, research showed that consumer satisfaction of food products and beverages when rated in controlled laboratory settings generally underestimates product acceptance when compared to real-life testing [32, 35–38].

Although the results from this open-label study are promising, future studies in a controlled laboratory setting should confirm these findings, evaluating After-Effect^©^ in double-blind, placebo-controlled clinical trials. In addition to examining the efficacy of After-Effect^©^, these studies preferably assess blood, saliva, and urine parameters to examine the possible mechanism of action of this new hangover cure. Examining also other potential hangover treatments in these clinical trials, preferably if they have other proposed mechanisms of action to reduce hangover severity, will further help researchers to elucidate the pathology of alcohol hangover. Also, it is important to incorporate cognitive and psychometric tests to determine if After-Effect^©^ is effective in reducing hangover-related performance on skills and abilities that are essential in daily activities such as driving a car or on-the-job performance. Finally, it can be determined if it is essential to take After-Effect^©^ before and after a drinking session. If it turns out that After-Effect^©^ is equally effective when taken only after alcohol consumption this should have great advantages. With the current formula of After-Effect^©^, consumers have to determine beforehand if they will engage in a drinking session that may produce hangover symptoms, while in real life heavy drinking is not always a planned activity. A French online survey among 4000 people revealed that almost half of those who acknowledge using anti-hangover products (48.8% of *N* = 991) prefer using the product *after* drinking alcohol, that is, before going to bed or the following day during hangover (Deenox, data on file). Only 22.3% prefer using the antihangover product before or during alcohol consumption. Therefore, future clinical trials should examine the effectiveness of After-Effect^©^ when taken *after* alcohol consumption only.

Also of interest would be to conduct dose-ranging studies. Currently, five capsules of After-Effect^©^ have to be taken. Since this was based on scientific literature on the effectiveness of individual ingredients it can be imagined that a reduction of the number of capsules to be taken (and thus the overall dosage of the ingredients) may sort the same effectiveness. In terms of potential adverse effects, but also with regard to user friendliness, it would be an advantage if less than 5 capsules would be sufficient to reduce hangover severity.

Taken together, the results from this first study on the effectiveness of After-Effect^©^ are promising and suggest that After-Effect^©^ may effectively reduce hangover symptom severity. This should, however, be verified and confirmed by placebo-controlled clinical trials.

## Figures and Tables

**Table 1:** Rationale for the ingredients included in After-Effect^©^.

Ingredients	Dose^1^	Effect	Improved symptoms^2^	Reference
Borago seeds oil (22% GLA)	1500 mg	Precursor of prostaglandin1	Headache, laziness, being tired	[18]
Magnesium	56.25 mg	Might be deficient	Withdrawal symptoms	[19, 20]
			Headaches	[21]
B6 vitamin	2 mg	Role in immune system	Uncomfortable feeling	[22]
B1 vitamin	4.2 mg	Might be deficient	Impatience, restlessness	[22, 23]
C vitamin	120 mg	Antioxidative properties	—	[24]
Fish oil (18% EPA, 12% DHA)	157.5 mg	Anti-IL1 effect	—	[25]
		Pro-inflammatory cytokines↓	—	[26]
*Silybum marianum * (2% silymarin)	80 mg	Hepatoprotective properties	—	[27–29]
*Opuntia ficus indica * (polar extract)	60 mg	Anti-oxidative properties	—	[24]

^1^Total dose of 5 capsules. ^2^Only those symptoms that showed a significant improvement during alcohol hangover are listed. GLA: gamma-linolenic acid, EPA: eicosapentaenoic acid (EPA), and DHA: docosahexaenoic acid.

**Table 2:** Hangover symptom scores when treated with After-Effect^©^, and expected scores if After-Effect^©^ had not been used (*N* = 103).

Hangover symptom	After-Effect^©^	No treatment	Significance
	Mean (SD)	Mean (SD)	
Thirst	3.96 (2.5)	6.62 (2.4)	*P* = 0.0001
Tired	4.34 (2.6)	6.96 (2.0)	*P* = 0.0001
Headache	2.71 (2.5)	6.42 (2.5)	*P* = 0.0001
Dizziness	1.34 (2.0)	3.35 (2.9)	*P* = 0.0001
Loss of appetite	1.79 (2.3)	4.39 (3.1)	*P* = 0.0001
Stomachache	1.60 (2.2)	4.57 (3.2)	*P* = 0.0001
Nausea	1.50 (2.1)	4.81 (3.0)	*P* = 0.0001
Heart racing	1.20 (1.9)	2.97 (2.9)	*P* = 0.0001
Global hangover severity	2.51 (2.0)	6.54 (2.6)	*P* = 0.0001
AHS total (mean) score	2.33 (1.6)	5.18 (1.9)	*P* = 0.0001

Scores range from 0 (absent) to 10 (extreme). Differences are significant if *P* < 0.05.

**Table 3:** Reported consumer satisfaction on the efficacy of After-Effect^©^ to reduce hangover symptoms.

Hangover symptom			After-Effect^©^ not effective (*N* = 12)		After-Effect^©^ effective (*N* = 91)	
	After-Effect^©^ helps?		After-Effect^©^	No treatment	After-Effect^©^	No treatment
	No	Yes	Mean (SD)	Mean (SD)	Mean (SD)	Mean (SD)
Thirst	27.2%	72.8%^∗^	6.4 (1.5)^†^	6.8 (1.7)	3.6 (2.4)^‡^	6.6 (2.5)
Tired	28.2%	71.8%^∗^	6.3 (2.5)^†‡^	7.4 (1.4)	4.1 (2.5)^‡^	6.9 (2.0)
Headache	18.4%	81.6%^∗^	5.1 (2.7)^†^	6.5 (3.0)	2.4 (2.3)^‡^	6.4 (2.4)
Dizziness	29.1%	70.9%^∗^	2.9 (3.1)^†‡^	3.7 (3.5)	1.1 (1.7)^‡^	3.3 (2.9)
Loss of appetite	38.8%	61.2%^∗^	4.1 (3.1)^†^	4.6 (3.4)	1.5 (2.0)^‡^	4.4 (3.1)
Stomach ache	31.1%	68.9%^∗^	3.1 (2.9)^†‡^	5.1 (3.1)	1.4 (2.0)^‡^	4.5 (3.3)
Nausea	23.3%	76.7%^∗^	3.8 (3.3)^†‡^	5.3 (3.4)	1.2 (1.7)^‡^	4.7 (3.0)
Heart racing	42.7%	57.3%	2.2 (2.8)	2.6 (3.1)	1.1 (1.7)^‡^	3.0 (2.9)
Global hangover severity	13.6%	86.4%^∗^	5.1 (2.7)^†^	6.0 (2.5)	2.2 (1.6)^‡^	6.6 (2.6)
Mean AHS score			4.3 (1.9)^†‡^	5.3 (2.0)	2.1 (1.4)^‡^	5.2 (1.9)
Concentration problems	34.0%	66.0%^∗^	5.3 (2.2)^†^	5.8 (2.6)	1.3 (1.7)^‡^	4.4 (3.1)

Significant differences (*P* < 0.05) in percentages of subjects who reported After-Effect^©^ is effective or not are indicated by ^∗^.

Significant differences (*P* < 0.05) in hangover symptom severity after using After-Effect^©^ or no treatment is indicated by ^‡^.

Significant differences (*P* < 0.05) in hangover symptom severity between subjects who reported After-Effect^©^ is effective or not are indicated by ^†^.

## References

[1] Verster JC, Herwijnen JV, Olivier B, Kahler CW (2009). Validation of the Dutch version of the brief young adult alcohol consequences questionnaire (B-YAACQ). *Addictive Behaviors*.

[2] Howland J, Rohsenow DJ, Edwards EM (2008). Are some drinkers resistant to hangover? A literature review. *Current Drug Abuse Reviews*.

[3] Verster JC, Stephens R, Penning R (2010). The alcohol hangover research group consensus statement on best practice in alcohol hangover research. *Current Drug Abuse Reviews*.

[4] Pittler MH, Verster JC, Ernst E (2005). Interventions for preventing or treating alcohol hangover: systematic review of randomised controlled trials. *British Medical Journal*.

[5] Verster JC, Penning R (2010). Treatment and prevention of alcohol hangover. *Current Drug Abuse Reviews*.

[6] Kaivola S, Parantainen J, Osterman T, Timonen H (1983). Hangover headache and prostaglandins: prophylactic treatment with tolfenamic acid. *Cephalalgia*.

[7] Wiese J, McPherson S, Odden MC, Shlipak MG (2004). Effect of Opuntia ficus indica on symptoms of the alcohol hangover. *Archives of Internal Medicine*.

[8] Penning R, van Nuland M, Fliervoet LAL, Olivier B, Verster JC (2010). The pathology of alcohol hangover. *Current Drug Abuse Reviews*.

[9] Kim DJ, Kim W, Yoon SJ (2003). Effects of alcohol hangover on cytokine production in healthy subjects. *Alcohol*.

[10] Verster JC (2008). The alcohol hangover—a puzzling phenomenon. *Alcohol and Alcoholism*.

[11] Mallett KA, Lee CM, Neighbors C, Larimer ME, Turrisi R (2006). Do we learn from our mistakes? An examination of the impact of negative alcohol-related consequences on college students’ drinking patterns and perceptions. *Journal of Studies on Alcohol*.

[12] Srikantha Murthy KR (2008). *Illustrated Suśruta Samhitā*.

[13] Stephens R, Verster JC (2010). The importance of raising the profile of alcohol hangover research. *Current Drug Abuse Reviews*.

[14] Frone MR (2006). Prevalence and distribution of alcohol use and impairment in the workplace: a U.S. national survey. *Journal of Studies on Alcohol*.

[15] Gjerde H, Christophersen AS, Moan IS (2010). Use of alcohol and drugs by Norwegian employees: a pilot study using questionnaires and analysis of oral fluid. *Journal of Occupational Medicine and Toxicology*.

[16] Kim J, Chung W, Lee S, Park C (2010). Estimating the socioeconomic costs of alcohol drinking among adolescents in Korea. *Journal of Preventive Medicine and Public Health*.

[17] Verster JC (2007). Alcohol hangover effects on driving and flying. *International Journal on Disability and Human Development*.

[18] Moesgaard S, Hansen NV *GLA effectively reduces hangovers. Double blind pilot study demonstrating the effect of a gamma-linolenic acid supplement (“Bio-Glandin 25”) on the after-effects of ordinary social drinking*.

[19] Mendelson JH, Ogata M, Mello NK (1969). Effects of alcohol ingestion and withdrawal on magnesium states of alcoholics: clinical and experimental findings. *Annals of the New York Academy of Sciences*.

[20] Min JA, Lee KS, Kim DJ (2010). The application of minerals in managing alcohol hangover: a preliminary review. *Current Drug Abuse Reviews*.

[21] Altura BM, Altura BT (1999). Association of alcohol in brain injury, headaches, and stroke with brain-tissue and serum levels of ionized magnesium: a review of recent findings and mechanisms of action. *Alcohol*.

[22] Laas I (1999). A double-blind placebo-controlled study on the effects of Morning Fit on hangover symptoms after a high level of alcohol consumption in healthy volunteers. *Journal of Clinical Research*.

[23] Li SF, Jacob J, Feng J, Kulkarni M (2008). Vitamin deficiencies in acutely intoxicated patients in the ED. *American Journal of Emergency Medicine*.

[24] Tesoriere L, Butera D, Pintaudi AM, Allegra M, Livrea MA (2004). Supplementation with cactus pear (Opuntia ficus-indica) fruit decreases oxidative stress in healthy humans: a comparative study with vitamin C. *American Journal of Clinical Nutrition*.

[25] Song C, Li X, Leonard BE, Horrobin DF (2003). Effects of dietary n-3 or n-6 fatty acids on interleukin-1*β*-induced anxiety, stress, and inflammatory responses in rats. *Journal of Lipid Research*.

[26] Mullen A, Loscher CE, Roche HM (2010). Anti-inflammatory effects of EPA and DHA are dependent upon time and dose-response elements associated with LPS stimulation in THP-1-derived macrophages. *Journal of Nutritional Biochemistry*.

[27] Pradhan SC, Girish C (2006). Hepatoprotective herbal drug, silymarin from experimental pharmacology to clinical medicine. *Indian Journal of Medical Research*.

[28] Wellington K, Adis BJ (2001). Silymarin: a review of its clinical properties in the management of hepatic disorders. *BioDrugs*.

[29] El-Kamary SS, Shardell MD, Abdel-Hamid M (2009). A randomized controlled trial to assess the safety and efficacy of silymarin on symptoms, signs and biomarkers of acute hepatitis. *Phytomedicine*.

[30] McKinney A, Coyle K (2004). Next day effects of a normal night’s drinking on memory and psychomotor performance. *Alcohol and Alcoholism*.

[31] McKinney A, Coyle K (2006). Alcohol hangover effects on measures of affect the morning after a normal night’s drinking. *Alcohol and Alcoholism*.

[32] Boutrolle I, Delarue J, Arranz D, Rogeaux M, Köster EP (2007). Central location test versus home use test: contrasting results depending on product type. *Food Quality and Preference*.

[33] Rohsenow DJ, Howland J, Minsky SJ, Greece J, Almeida A, Roehrs TA (2007). The Acute Hangover Scale: a new measure of immediate hangover symptoms. *Addictive Behaviors*.

[34] Penning R, McKinney A, Verster JC (2012). Alcohol hangover symptoms and their contribution to overall hangover severity. *Alcohol Alcoholism*.

[35] Meiselman HL, de Graaf C, Lesher LL (2000). The effects of variety and monotony on food acceptance and intake at a midday meal. *Physiology and Behavior*.

[36] Hersleth M, Mevik BH, Næs T, Guinard JX (2003). Effect of contextual factors on liking for wine—use of robust design methodology. *Food Quality and Preference*.

[37] Kozlowska K, Jeruszka M, Matuszewska I, Roszkowski W, Barylko-Pikielna N, Brzozowska A (2003). Hedonic tests in different locations as predictors of apple juice consumption at home in elderly and young subjects. *Food Quality and Preference*.

[38] King SC, Weber AJ, Meiselman HL, Lv N (2004). The effect of meal situation, social interaction, physical environment and choice on food acceptability. *Food Quality and Preference*.

